# Selections

**Published:** 1816-10

**Authors:** 


### PART III.

### SELECTIONS.

—— Factorum est copia nobis.

### FRENCH DICTIONARY OF MEDICAL SCIENCES.*

( Continued from page 81. )

*Medical Jurisprudence.—Article Aliéné*, from the Latin *alienus* (an insane person, one beside himself.) A lunatic, incapable of appreciating the morality and consequences of his actions, is not responsible for any outrage which he may commit against the social order. He cannot exercise his civil rights, nor be compelled to fill the offices imposed by the state on other citizens. The abuses which may result from false application of these exceptions, the personal safety of the lunatic, and public security, demand medical investigation to ascertain whether the state of insanity be real; and this falls within the province of the juridical physician. Again, it belongs to medical police to combine and execute means which may improve the condition of the lunatic, and prevent the occurrence of injury to himself or others.

Insanity requires the decision of the judicial physician only when it is doubtful; and there is more difficulty in deciding upon its reality when an individual is interested in feigning or concealing this state, than when it is imputed to him. On this subject, the following general precepts may be adopted: 1st. Can any interested motive be suspected, on the part of the patient, or others, to feign, conceal, or impute madness? 2dly. The characters, displayed in the individual case should be determined by rigorous analysis, and the species of derangement to which it belongs: This is an important rule, as it should constitute the principal basis of observation. In truth, if madness be imputed against the will of the patient, the physician will always readily determine, whether the pretended lunatic exhibits in his actions or discourse, any traces of

* The regular analysis of the articles on Medical Jurisprudence, continued in the French Dictionary is here commenced They are the production of Marc, a writer eminently qualified to do justice to this important subject. We shall occasionally quote from the works of other French, German, and British authors; particularly on obscure points requiring illustration; and carefully point out any peculiar views which may be taken of the subject under discussion, in the system of English legislation, and state the general principles by which its decisions are guided.—Editors.

it. If it be affected, the impostor will with difficulty avoid committing some inconsistences with respect to the peculiar kind of madness, which must lead to detection. 3dly. It should be ascertained whether the causes of the intellectual derangement correspond with the affection. The conformation of the cranium, of the other organs of the senses and of speech should be attentively examined: nevertheless, inferences are not to be rashly drawn from the state of these organs, Idiotism, for instance, especially when acquired, may occur in an individual whose head possesses the regular configuration. A small or partially mis-shapen head does not invariably preclude the integrity of the intellectual faculties. Thus the vices of conformation should only be regarded collectively with other signs. 4thly. It is essential to gain' precise information as to the period at which the disease began. The existence of it will acquire probability, if the patient have committed acts of insanity previously to the time at which circumstances might warrant a suspicion of interested motives, on the part of himself or others. So, for example, a criminal, whose intellect became deranged after he fell into the hands of justice, would afford stronger grounds of doubt, than he who betrayed signs of insanity, while yet at liberty. Finally, menaces and ill-treatment should, as much as possible, be banished from our researches. Such means are, at most, only admissible, when they complete an imposing mass of data by which fraud is brought to light. It is of importance to recollect that sudden and powerful excitement of the passions has sometimes immediately effected a cure;* and that, in this event, we are not to infer the non-existence of madness.

Mental derangement may be acute or chronic. By the former is meant that, which, dependent on transient causes, is limited to a transient excess; and which, once terminated, does not recur. Such are the acute delirium of fever, intoxication, and the delirium induced by vegetable poisons. These three states enjoy a certain rank in the history of simulated diseases. Thus, in an individual, who has been ill-treated, an accidental malady may supervene: the patient, from a vindictive spirit, wishes to appear worse than he really is; and raves, in order that the danger may be attributed to the injuries which he has received. This case more frequently occurring than is supposed, requires great penetration on the part of the physician; who can distinguish it only as he seizes the relations connecting the phænomena of which the diagnosis is composed, and as he appreciates the efficacy of the causes alleged by the patient or others. We may also, in the way of experiment, propose unpleasant or painful means of cure, and observe if the proposal produce on the pulse, features, or com-

* We have known a person, who had been for some years insane, become instantaneously and permanently calm and rational, upon the unexpected view of the body of a deceased and beloved relative.—Edit.

plexion of the patient, any alteration, indicating fear.* There are no certain means of distinguishing real from pretended intoxication, especially in those who possess to high perfection the art of counterfeiting it, by the aid of a slight excess of intoxicating liquor, not sufficient to disorder the intellect. This, perhaps, partly explains why, in many countries, the laws establish no great difference between crimes committed during a state of intoxication or otherwise. That state is most commonly the subject of juridical research only when it no longer exists. We cannot then hope to decide it except by common testimony, which falls less within the province of the physician than the lawyer. The delirium, induced by poisons, may serve either for the feigning or imputation of periodical mania, to which we shall hereafter advert. But besides that narcotic poisons determine peculiar symptoms, readily discriminated by the physician, the delirium resulting from their use is of short duration: it is, moreover, if the dose have been strong, followed by disorder of the nervous action, and by comatose affections, not equally observed in periodical mania.

Authors, who have written on chronic mental derangement, have not hitherto been able to establish characters sufficiently decided to determine its numerous shades. Thus, the treatises on forensic medicine comprehend only three principal kinds of the disease, delirium, melancholy, and fatuity. Professor Pinel has more accurately divided mental alienation into five different kinds ; and this division we shall follow, after having said something on the causes of insanity, which it behoves the juridical physician to appreciate.

The proximate causes of chronic madness, for the most part, elude our research : not so with the remote or external causes, which etiological examination often enables us to discover. The essential points on which it should be directed, are these: *Hereditary disposition ;* this constitutes one of the strongest presumptions as to the existence of insanity.—*Temperament.* A dry irritable temperament disposes, as we know, to melancholic affec-

* The application and value of such experiments will be well illustrated by the following fact: A vagrant, apparently the victim of idiotism and epilepsy, after a violent paroxysm of convulsion, fell into insensibility. He could neither take food, nor articulate: nothing could arouse him. Yet many circumstances conspired to render him an object of suspicion. A consultation of medical gentlemen was held in the chamber; and, at length, one of them announced, formally and in a loud voice, that, unless the symptoms had disappeared by the ensuing morning, it would be absolutely necessary to scalp and trepan the patient's skull. Orders, at the same time, were given to prepare the necessary instruments at a certain hour. The patient was observed to listen attentively to the formidable harangue. Morning came: and not only the symptoms, but their subject, had disappeared. The panic-stricken rogue had escaped from a window, through which it was hardly imagined that a child could have passed, leaving all his ill-gotten treasure behind him, and a greater part of the hair of his head, upon the bars.—EDITORS.

tions.—*Mode of life.* A knowledge of this may throw some light on the state of the intellectual powers. Thus, profound thought, mental conflicts, an inactive and monotonous life, excessive labour, especially in a dry and warm atmosphere, or sedentary occupations in a painful or unnatural posture, may operate in deranging the intellect.—*Education.* Its influence on the direction of our thought is well known; and again, on our passions, which are among the most frequent causes of insanity. Men most commonly lose their reason from ambition; women, from jealousy; girls, from love: and the actions and conversation of the lunatic generally preserve a striking character of the ruling passion, which may direct the juridical physician.—*Climateric influence.* The inhabitants of warm countries display a greater nervous mobility than those of cold; and are naturally more subject than the latter to the species of insanity resulting from exalted imagination. Excessive cold, a moist and cloudy atmosphere, may equally impair the intellectual faculties; but this effect is almost always the inverse of that produced by heat. The latter unduly exalts the perceptions; the former impairs them. These principles will serve, with other signs, to expose the nature and existence of insanity. Thus mania may be generally suspected in the native or inhabitant of a hot climate; melancholy, in those of a wet and cloudy region; idiotism, in persons long exposed to severe colds: yet certain organic impressions, dependent on climateric influence, remain, when the individual is no longer exposed to it. In the examination of these general causes should be comprised the numerous circumstances which may precede, or accompany, the loss of reason.

The office of the juridical physician consists principally in their just appreciation; and by these views should his researches be directed. Thus, in idiotism, the precept of Zacchias is to be observed; who considers cowardice and submission as the badge of idiots: yet sudden provocations sometimes excite in them a transient burst of rage, resembling maniacal delirium. The memory and conception of a suspected idiot should lastly be put to the proof; and all experiments of this kind so conducted, that the subject of them may not be conscious of the end for which they are instituted.

The two opposite forms, which melancholy and exclusive delirium present, the one consisting of a sort of self-sufficiency, the other of a profound and fearful dejection, merit all the attention of the physician; for these two forms probably never co-exist; and yet this complication may be observed in the feigned melancholic. In all cases, the real maniac and melancholic are moved only by objects connected with the subject of insanity. Individuals, really affected with madness, particularly the furious and melancholic, are subject to the different passions in a very vague and uncertain manner, no way relative to the circumstances in which they are placed; and this principle should direct our moral experiments. The real melancholic is strongly prejudiced in favour of his own opinions; the least contradiction displeases him: but the pretender neglects this essential point of character, if the contradiction be skilfully conducted; and the silence peculiar to the first, is often wanting in the latter. The complaints of these, when sure of being seen or heard, their repugnance to gloomy situations, are not, at least equally, observed in the real sufferer. His sleep is restless and interrupted, while that of the impostor is sound. Some melancholics, like certain maniacs, are little sensible to the action of drastic purgatives. This sign, by itself insufficient, may have some weight in combination with others. The same may be said of certain external characters, which commonly distinguish maniacs and melancholics, and denote a dry and irritable temperament, as quickness of eye, turgid veins, black or red hair, hepatic complexion, emaciation.

*Mania with delirium* is, of all the forms of insanity, most easy to appreciate. They who have read its description by Pinel,* cannot mistake it.

*Dementia or abolition* of thought displays equally a decided character. The rapid uninterrupted succession of isolated ideas, and light extravagant emotions, render it easy to be feigned: but here the experiment, already spoken of, will apply, which consists in exciting a moral emotion; this will have no influence, or merely a very slight one, if the malady be not affected. Again, however able an impostor may be, we may always remark a sort of hesitation or reflection in his discourse: the extravagant ideas flow not with the same rapidity as in the real madman. Another trial might be instituted by making the patient repeat a series of ideas recently delivered. The pretender, instead of deviating, would think fit to reiterate the same words in proof of his insanity.

There is no kind of insanity which more strongly claims the attention of the physician and legislator than *mania without delirium.* Scarcely known till the time of Pinel, often misunderstood or neglected, it has led to the scaffold many deplorable victims who merited commiseration rather than punishment. Unhappily, there exists no other mean of proving this dreadful state, in which an instinct, irresistible as destructive, impels the maniac to actions the most revolting, but long confinement, during which he may be observed in the moment of seizure by his noxious propensity. Then, if it be real, we shall remark extreme agitation, redness of the face, sparkling of the eyes, and, perhaps, as in the propensity to suicide, a higher temperature of the hypochondria. Women are, in general, more prone than men to this kind of mania, especially during menstruation, if any morbid condition exist, or in pregnancy: these different states demand peculiar consideration. Moreover, the moral circumstances which precede or accompany a crime, almost always indicate whether it be the result of a vicious disposition or deranged intellect. With the real criminal, there inva-

* *Traité Médico-Philosophique sur l'Aliénation Mentale.* Paris, 1809.

riably exists some interested motive: thus murder, followed by theft, can never be attributed to mania.

Something remains to be said of certain species of insanity, which frequently interest the juridical physician; to wit, *nostalgia*, *ecstacy*, and *demonomania*. We may apply to them what has been generally said on exclusive delirium. Thus, the real nostalgic is distinguished from the pretender by a certain reserve on the cause of his sadness. This disease, moreover, induces depression and slowness of pulse, decomposition of the physiognomy, difficult and spasmodic respiration, protracted anorexy, and rapid emaciation; symptoms which cannot be feigned. Ecstacy and demonomania are, in many cases, the result of alienation, which may be distinguished by its peculiar characters: otherwise they are assumed for fraudulent purposes. But the impostor combines accessory phænomena, which lead to his detection: such as extravagant contortions, excretion or ingestion of strange substances. The real ecstatic and demonomaniac are proper subjects for confinement. The arm of justice will most effectually exorcise the pretender.

It is needless to remark, how greatly the periodic character aggravates the difficulty of decision in insanity, whether feigned, concealed, or imputed. The only effectual means is to submit the patient to a long and rigorous watch, in order not only to witness a paroxysm, but to prevent its artificial excitement by the ingestion of certain poisons. We should particularly ascertain whether the disease be regular, or otherwise, in its recurrence. Dementia and idiotism are rarely periodical; melancholia most commonly is so: mania with delirium, almost always; mania without delirium, invariably. Vigilance should be redoubled at the period when the madness will probably recur. The approach of tempests produces on insane persons, of every description, a sort of effervescence: hence electricity and Galvanism may probably be turned to account in these researches. But as periodic insanity is sometimes independent of the influence of the seasons, the attention of the juridical physician should be directed to the causes capable of determining the paroxysm, and their value should be justly appreciated. We may, lastly, observe, that in melancholy, and yet more in periodic mania, the patient has generally, during the lucid interval, no idea, or only a faint one, of the paroxysm: hence he broods in silence over his condition, and likes not to have it spoken of. The conduct of the pretended madman is different: he tries to excite the compassion of those who visit him.

Of the situation best calculated for the erection of a lunatic asylum; of the principles on which, with regard to safety, convenience, and salubrity, its structure should be directed; of the vigorous, humane, and enlightened policy which should preside over and regulate its internal œconomy; of the necessity of a rigorous system of inspection, on the part of Government, to prevent the dreadful abuses by which these institutions are too often rendered the curse of humanity; much might be said: but we shall conclude, for the present, with the following reflection of the French writer:

—" In the eastern countries, far inferior to our own in civilization, the lunatic is treated with that respect which the contemplation of misfortunes inspires in every feeling heart. No one is allowed to cast the slightest ridicule upon the state of degradation to which the unfortunate creature is reduced. Why do we not imitate an example so affecting? Why do we longer suffer lunatics, whose harmless insanity renders confinement unnecessary, to be hourly exposed, in country towns and villages, to the insults and mockery of a thoughtless mob? Why not repress abuses, hostile alike to the individual on whom they are inflicted and to sound morality?"

The distinction set up by Foderé between melancholia and hypochondriasis is at once so judicious and correct, that we shall transcribe it. In common writings and conversation, these two forms of mental affection are confounded.—" On first view, the melancholic and hypochondriac exhibit features of resemblance; inasmuch as both are influenced by fear respecting an illusory object, and incessantly cherish suspicion and distrust, for the most part, of their relatives and best friends: but they differ in the following circumstances. 1st. The habit of body of the melancholic is opposite to that of the hypochondriac. 2dly. The fear of the melancholic is accompanied by reserve, prudence, and secrecy: it destroys not his courage. On the contrary, that of the hypochondriac is weak, variable, credulous, and confiding. The illusion of the melancholic is more relative to the moral than physical condition. He believes, for instance, that he is encompassed by rogues, that the world has conspired to vilify or ruin him. Rarely is the fear of corporeal destruction the subject of his delirium: in fact, he is often on the brink of suicide. The central point of delirium of the hypochondriac is exclusively his health; and the idea of impending destruction is that which engrosses all his faculties and conversation. There is a somewhat of the noble and generous in the first; the latter is completely selfish."* Suicide, he considers as invariably the act of madness.

By the existing laws of this country, " an ideot or natural fool" is defined to be " one who has so little sense as to be unable to number to twenty, or to tell his age, or to answer any common questions," and " has not reason to discern what is to his advantage or disadvantage." But a person who ordinarily possesses as much knowledge and judgement as an infant fourteen years old, is in a state to be found guilty of treason and felony.—Again, supposing an individual to suffer a daily paroxysm of madness, he is responsible for any crime which he may commit during the lucid interval.

Crimes, perpetrated under the influence of ungovernable rage, subject the offender to the vengeance of the law.† It is the same with regard to intoxication, if this state have been voluntarily induced, and not brought on by medicine unseasonably given, or poison accidentally swallowed.‡

---

* *Traité de Médicine legale.*

† Dr. Male's Epitome. ‡ Sir M. Hale.

"A lunatic, though not punishable for criminal offences, is, nevertheless, compellable, in a civil action, to give satisfaction for damages. Though he labours under many legal disabilities, he is allowed to act in some things by his committee, and even to sue for a divorce from his wife, by reason of adultery."*

Lastly, the idiotic or insane are incapable of bequeathing property; but, "a melancholic person may make a will, if it can be proved that he has 'a disposing memory,' so as to be able to make a disposition of his estate with understanding and reason.†"

The monographs on insanity, in its relation with juridical research, mentioned in the French Dictionary, are those of *Bose*, 4to. Leipsic, 1774; *Gruner*, 4to. Jena, 1783; *Platnar*, Part III, 4to. Leipsic, 1796-7.—On this subject may also be consulted with advantage, the curious *Illustrations of Madness*, by Haslam, London, 1810; the Works of Mahon and Foderé; the excellent, but concise *Epitome* of Dr. Male,‡ and the article *Medicina Forensis*, in the *London Medical Dictionary*.—For a particular account of the causes, symptoms, and treatment of insanity, we refer the reader to the works professedly written on that subject, as those of Arnold, Crichton, Pinel, Cox, Haslam, Crowther, and Hill.

EDITORS.

### FOREIGN JOURNALS.

I. PATHOLOGY OF THE BRAIN.—"*Case of Fungus developed in the Substance of the Brain, and adhering to the internal surface of the Dura Mater.*§

DORIFAT (the subject of the following history) kept a billiard table, and gave lessons in this game. He was of a strong constitution, and had hitherto enjoyed good health. Without evident cause, he was attacked by head-ach, dimness of sight, and a sensation of weight in the right arm, with difficulty of moving it. As, in the capacity of a vender of lemonade, he had lived, for ten years, in a vault, these symptoms were attributed to rheumatism; but the treatment, prescribed under this supposition, was unavailing. On the contrary, the voluntary motions of the right arm became gradually more difficult; and were, at length, utterly lost. The sen-

---

* Male † Same, page 9.

‡ Dr. Male, in stating the general morbid appearances which the brain of insane persons exhibits on dissection, observes, that, "a gritty matter, consisting of phosphate of lime, has been found in the pineal gland." The fact is, the *healthy* gland almost invariably contains this particle of phosphate.—EDIT.

§ *Sur un fongus developé dans l'epaisseur du cerveau, et adherent a la interne de la dure mère, par M. Hebreard, D. M. Bulletin de la Faculté de Medecine, &c.* 1816, *No. IV.*

sibility of the limb was also by degrees abolished. Nevertheless, the intellectual faculties yet continued unimpaired. The man had even acquired sufficient address with the left arm to continue his instructions in billiards.

"In the course of 1812, the right thigh and leg lost their sensibility. He walked with difficulty, but yet continued his employment. In about a year after the occurrence of the hemiplegia, he perceived that his vision was impaired, and experienced difficulty of articulation. His ideas also became evidently confused. He sank, at last, into a state of perfect idiocy, and was received into the Bicetre, as impotent and paralytic. He was then forty-two years old. His nutritive functions exhibited not the slightest derangement.

"On the 21st of January, 1816, he was brought to the hospital in the following condition:—hemiplegia and insensibility of the right side; loss of consciousness; slight spasmodic motions of the muscles of the face; incessant hiccough; eyes dim and staring vacantly; vision of both apparently destroyed; mouth open and drawn to the left; tongue red and little moist; cheeks flushed; sensibility of the left side impaired. The pulse regular and strong; heat natural. By means of tonics and stimulants, I succeeded in rousing my patient from this state of torpor. Ten days after his entrance, he seemed to comprehend some simple questions put to him, and replied in monosyllables. In the course of about twenty-five days, a moist heat manifested itself; and expectoration became thick and viscid. Yet the symptoms of cerebral congestion evinced no tendency to remit. He was always in the same state of drowsiness and immobility, although he could swallow liquids, broth, and even soup. In this situation he remained till the 7th of March. This period was marked by dryness of the tongue, and absolute and general loss of sensibility. The pulse grew small and intermittent; and the patient, at last, died, or rather became extinct, on the fourteenth.

"*Dissection.*—Before removing the calvaria, we perceived that the integuments and pericranium adhered strongly on the left parietal bone towards its anterior superior angle; yet no trace of cicatrix of the integuments could be distinguished: they were every where covered with hair. When this adhesion had been destroyed, we saw that the bone was deprived of its periosteum in this part, to the extent of a square inch. The external table was reddish and rugous; and the corresponding part of the internal was much more changed: it presented the same reddish colour, numerous asperities, and small cellular cavities, which received little fungous excrescences in the form of granulations; by means of which, the dura mater adhered strongly to the morbid portion of the parietal bone. The groove, which lodges the (middle) meningeal artery, and the calibre of the vessel itself, were considerably enlarged.

"The dura mater corresponding to the morbid point of the parietal bone, was much thicker than natural: in every other part it was sound. Below, was felt an indurated body; and on cutting the dura mater, we found a fungus, of irregular figure, as large as a fist. This fungus adhered to the internal surface of the dura mater by distinct fibres, which, uniting, formed a short pedicle about one inch in diameter. The fungous tumor was lodged in the middle lobe of the left hemisphere of the brain, above the corresponding lateral ventricle; from which it was separated by a very thin layer of medullary substance. The whole brain was flabby; and the part surrounding the tumour reduced to pulp. The pia mater and arachnoid membrane, corresponding to the tumour, were destroyed. The ventricles contained an unusual quantity of fluid. The tumour, on incision, displayed a substance of a yellowish grey colour, and tolerably firm, except in the centre, which was soft and even pulpy. Black blood issued in drops from the dilated vessels which traversed the tumour. It was enveloped, as in a cyst, by a membrane, which appeared to be of a fibrous structure, and furnished by the dura mater.*

"The fact which we have just recorded seems, in many respects, remarkable.—First, From the situation of the tumour on the internal surface of the dura mater, and amidst the substance of the brain.—Secondly, From the gradual abolition of the intellectual faculties, a phænomenon which apparently originated in compression of the brain by the increasing volume of the tumour.—Thirdly, From the developement of the meningeal artery, increased to twice its natural calibre. Lastly, From the death, which did not take place until long after the appearance of the early symptoms; nor till the compression, exercised on the brain by the tumour and effused fluid of the ventricles, had reached its utmost limit."

### PART IV.

### MEDICAL MISCELLANIES.

Quicquid agunt Homines.

*Cessation of the Pulse at both Wrists for Thirty Hours, with ultimate Recovery.*—One of the Editors was lately requested to see a woman who was reported to be very ill. He found her with some pyrexia, heat on the skin, great irritability of the stomach, inability to retain any food or drink, violent pain in the left hypo-

* This anatomical preparation was presented by Dr. Hébréard, to the Medical Society of Paris.

